# Calpastatin Overexpression Preserves Cognitive Function Following Seizures, While Maintaining Post-Injury Neurogenesis

**DOI:** 10.3389/fnmol.2017.00060

**Published:** 2017-03-23

**Authors:** Vanessa M. Machado, Ana Sofia Lourenço, Cláudia Florindo, Raquel Fernandes, Caetana M. Carvalho, Inês M. Araújo

**Affiliations:** ^1^Center for Neuroscience and Cell Biology, University of CoimbraCoimbra, Portugal; ^2^Center for Biomedical Research, CBMR, University of AlgarveFaro, Portugal; ^3^Regenerative Medicine Program, Department of Biomedical Sciences and Medicine, University of AlgarveFaro, Portugal; ^4^Algarve Biomedical Center, University of AlgarveFaro, Portugal

**Keywords:** calpains, calpastatin, cognition, hippocampus, post-injury neurogenesis

## Abstract

In the adult mammalian brain, new neurons continue to be produced throughout life in two main regions in the brain, the subgranular zone (SGZ) in the hippocampus and the subventricular zone in the walls of the lateral ventricles. Neural stem cells (NSCs) proliferate in these niches, and migrate as neuroblasts, to further differentiate in locations where new neurons are needed, either in normal or pathological conditions. However, the endogenous attempt of brain repair is not very efficient. Calpains are proteases known to be involved in neuronal damage and in cell proliferation, migration and differentiation of several cell types, though their effects on neurogenesis are not well known. Previous work by our group has shown that the absence of calpastatin (CAST), the endogenous inhibitor of calpains, impairs early stages of neurogenesis. Since the hippocampus is highly associated with learning and memory, we aimed to evaluate whether calpain inhibition would help improve cognitive recovery after lesion and efficiency of post-injury neurogenesis in this region. For that purpose, we used the kainic acid (KA) model of seizure-induced hippocampal lesion and mice overexpressing CAST. Selected cognitive tests were performed on the 3rd and 8th week after KA-induced lesion, and cell proliferation, migration and differentiation in the dentate gyrus (DG) of the hippocampus of adult mice were analyzed using specific markers. Cognitive recovery was evaluated by testing the animals for recognition, spatial and associative learning and memory. Cognitive function was preserved by CAST overexpression following seizures, while modulation of post-injury neurogenesis was similar to wild type (WT) mice. Calpain inhibition could still be potentially able to prevent the impairment in the formation of new neurons, given that the levels of calpain activity could be reduced under a certain threshold and other harmful effects from the pathological environment could also be controlled.

## Introduction

New neurons in the adult mammalian brain are known to originate from mainly two regions, the subventricular zone, in the walls of the lateral ventricles, and the subgranular zone (SGZ), in the dentate gyrus (DG) of the hippocampus (Bond et al., 2015). Neural stem cells (NSCs) from the subventricular zone migrate long distances, through a rostral migratory stream, into the olfactory bulb, differentiating into interneurons. NSCs from the SGZ, in turn, migrate shorter distances into the granular zone (GZ), where they fully mature into granule neurons after 2 months (Aimone et al., 2014; Jin, 2016). Functions of these newborn cells from both regions have been consistently associated with learning and memory (Deng et al., 2010; Lazarini and Lledo, 2011).

When a brain lesion occurs, behavioral traits and adult neurogenesis can become altered. In neurodegenerative diseases, neurogenesis is mostly hampered, but in acute disorders, such as temporal lobe epilepsy, NSC proliferation can increase, in a possible attempt to repair the damage. However, this repair is limited by impaired cell migration or decreased survival of new neurons (Kaneko and Sawamoto, 2009; Ma et al., 2009).

Another feature of brain damage is excitotoxicity, which leads to an increase of cellular levels of calcium, activating several proteases, including calpains (Neumar et al., 2001). Calpains are ubiquitously expressed calcium-activated proteases, with only one known natural endogenous inhibitor, a protein called calpastatin (CAST; Murachi, 1984). In the CNS, calpain inhibition has been shown to afford neuroprotection, showing improved neuronal function and limiting neuronal damage in several brain disorders (Saez et al., 2006). Although little is known about their contribution to neurogenesis, calpains were seen to be involved in cell proliferation, migration and differentiation in other systems (Rock et al., 2000; Yajima and Kawashima, 2002; Lokuta et al., 2003; Parnaud et al., 2005; Qiu et al., 2006; Shimada et al., 2008; Kashiwagi et al., 2010; Kuchay et al., 2012).

Previous studies by our group show that increased calpain activity, through lack of CAST, can impair early stages of neurogenesis in the hippocampus, the main structure involved in learning and memory (Machado et al., 2015). Moreover, the kainic acid (KA) model of seizure-induced hippocampal lesion presents excitotoxic damage mediated by calpains (Araújo et al., 2008) and is characterized by pathologic alterations in hippocampal neurogenesis (Carreira et al., 2010). With this work, we proposed to assess whether overexpression of CAST (hCAST mice) could improve cognitive recovery and neurogenesis after KA-induced hippocampal lesion.

## Materials and Methods

### Animals

Three-month old male and female hCAST mice (Rao et al., 2008), in a C57BL/6 background, and their wild type (WT) littermates, were used in this study. hCAST mice present CAST overexpression, due to the cloning of a human CAST construct (Hitomi et al., 1998) into a Thymocyte differentiation antigen 1.1 expression cassette (Thy1). The animals were kept in our animal facilities, in a room with controlled temperature (21 ± 1°C) and humidity (55%), with food and water *ad libitum* in a 12-h dark:light cycle. All experiments were performed in accordance with institutional and European guidelines (2010/63/EU) for the care and use of laboratory animals. The procedures performed in mice described in this work have been reviewed and approved by the Animal Welfare Body of the Center for Neuroscience and Cell Biology and have been approved by the Direcção Geral de Alimentação e Veterinária (reference 0421/000/000/2013).

### KA Treatment

Hippocampal damage was induced by administering KA subcutaneously (25 mg/kg, in a concentration of 5 mg/ml, in a saline (SAL) solution of 0.9% NaCl), as previously described by our group (Carreira et al., 2010). After KA administration, the animals went through several stages, according to a well-defined scale (Schauwecker and Steward, 1997): immobility (I), tail/forelimb extension/rigid posture (II), repetitive movements/head bobbing (III), rearing and falling (IV), continuous rearing and falling (V), severe tonic-clonic seizures (VI). Only mice that reached stage V or higher were used in this study. SAL-treated animals were used as controls.

### Behavior Analysis and Neuronal Differentiation

**Figure 1:**
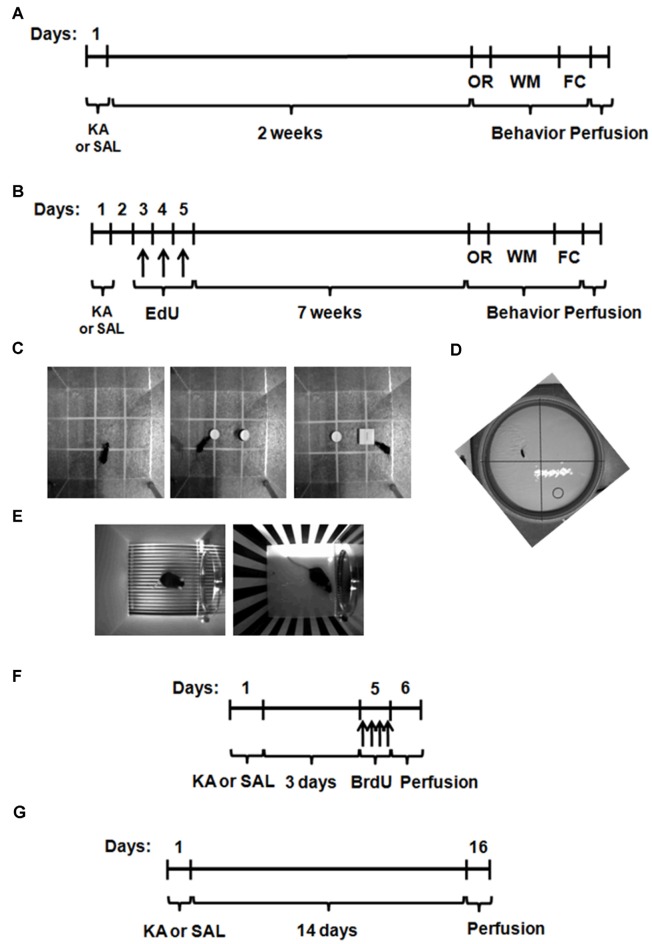
**Experimental procedures for the study of behavioral recovery and neurogenesis after kainic acid (KA) or saline (SAL) treatment.** Wild type (WT) and hCAST mice were treated with either SAL or KA (25 mg/kg, s.c.). **(A)** To study behavioral recovery in the short-term group, the cognitive behavior of the animals was tested on the 3rd week after treatment. **(B)** For the behavioral recovery in the long-term group and the study of neuronal differentiation, mice were given 5-ethynyl-2′-deoxyuridine (EdU; 50 mg/kg, i.p.) on days 3, 4 and 5, 8 weeks before sacrifice, and the cognitive behavior was tested during the last week. **(C)** On the first day, object recognition (OR) was tested. **(D)** Water maze (WM) trials were performed on the next 3 days and the final test, without the platform, was on day 4. **(E)** On the next day, fear conditioning (FC) trials were performed. One day later, the animals were at first left on the same context and, after 3 h, the context was completely modified and the cued test performed. The animals were sacrificed on the next day. **(F)** To study neural stem cell (NSC) proliferation, mice were given 5-bromo-2′-deoxyuridine (BrdU; four doses of 50 mg/kg, i.p.) on day 5, up to 12 h before sacrifice. **(G)** To study neuroblast migration, mice were sacrificed 14 days after treatment.

For the studies of short-term behavioral recovery, three different behavioral tests were performed in WT and hCAST mice, on the 3rd week after KA or SAL treatment (Figure 1A): object recognition (OR; Figure 1C), water maze (WM; Figure 1D) and fear conditioning (FC; Figure 1E), as explained further in more detail. To assess long-term behavioral recovery and neuronal differentiation after lesion, WT and hCAST mice were treated as shown in Figure 1B. The thymidine analog 5-ethynyl-2′-deoxyuridine (EdU) was intraperitoneally administered to WT and hCAST mice on days 3, 4 and 5 after KA or SAL treatment, and the animals were sacrificed by transcardial perfusion after 8 weeks, when new neurons are fully mature (Aimone et al., 2014). Behavioral analysis was performed during the last week.

### Object Recognition

For the study of recognition memory, the OR test (Bevins and Besheer, 2006) was used. Mice were presented to two similar objects, following habituation to the apparatus (40 × 40 × 40 cm^3^ acrylic box), after which one of the objects was replaced by a novel one. Each stage of the test had the duration of 5 min, 2 h apart, and the apparatus and objects were cleaned with 10% ethanol between animals. In the presence of the objects, the percentage of time spent with each object (totalizing 100%) was measured. The analysis was done in videos acquired with a GoPro Hero (GoPro, Inc., San Mateo, CA, USA) during testing of 3–5 animals per group (for short-term recovery) and 12–15 animals per group (for long-term recovery), using the Any-maze software (version 4.99, Stoelting Co., Wood Dale, IL, USA).

### Morris Water Maze

To evaluate spatial learning and memory, the WM test (Morris et al., 1982) was performed. During the first 3 days, the animals were trained to find a platform (10 cm diameter, carved to increase grip) hidden 0.5 cm below the water surface level in a circular pool (1.52 m diameter), in a fixed location. Visual cues on the walls around the pool were provided to help the mice locating the platform and the water temperature was maintained at 22°C ± 1°C. The pool was divided into four quadrants and, on each day of training, the animals were placed in a different quadrant, always facing the border of the pool, under the course of four 60 s trials, 30 min apart. The latency to escape was measured as the time each animal took to find and climb onto the platform. If the animal did not reach the platform within the course of the trial, the latency to escape was considered 60 s and the animal was left on the platform for 10 s, in order to learn the platform location. On the last day of testing, the platform was removed and the mice were placed in the quadrant opposite to the target quadrant (where the platform was previously located), facing the border of the pool. The time spent on each quadrant and the number of crossings over the previous platform location were measured for 60 s, to evaluate if the animals remembered where the platform was located during training. All animals were dried under an infrared light after each trial. The analysis was done in videos acquired with a GoPro Hero (GoPro, Inc., San Mateo, CA, USA) during testing of 4–5 animals per group (for short-term recovery) and 11–16 animals per group (for long-term recovery), using the Any-maze software (version 4.99, Stoelting Co., Wood Dale, IL, USA).

### Fear Conditioning

Associative learning and memory were tested by performing the FC test (Wehner and Radcliffe, 2004). The first day of testing consisted of learning to associate a cue tone (80 dB) with a footshock (0.7 mA). For this part of the test, mice were placed in a chamber (17 × 17 × 25 cm^3^) with a grid floor inside a soundproof box (Stoelting Co., Wood Dale, IL, USA), with a background white noise, 70% ethanol scent and 100% lux lighting (context A). After 2 min (acquisition/habituation), the tone was played for 30 s and the footshock given during the last 2 s of tone. This was repeated two more times, after a 60 s rest. On the next day, the association of the context with the footshock was tested (context test), by placing the mice inside the chamber in the same conditions as during learning for 5 min, without presentation of tone or footshock. After 3 h, the association of the footshock with the tone (cued test) was tested, by placing the animals in a completely different environment for 3 min (habituation to new context) and then playing the tone for the last 3 min. The new context consisted of a background fan noise, vanilla scent, 10% lux lighting, and the previously used chamber was altered by using striped black and white walls and a white floor covering the grid (context B). During the whole FC testing, the chamber was cleaned with 10% ethanol between animals, and the fear behavior measured by calculating the percentage of freezing time, for 3–5 animals per group (for short-term recovery) and 9–16 animals per group (for long-term recovery), using the Any-maze software (version 4.99, Stoelting Co., Wood Dale, IL, USA).

### NSC Proliferation

Cell proliferation after KA or SAL treatment was assessed by the incorporation of the thymidine analog 5-bromo-2′-deoxyuridine (BrdU), as shown in Figure 1F. WT and hCAST mice were given either KA or SAL (9–14 animals per group) and BrdU was administered intraperitoneally (four doses of 50 mg/kg, 2 h apart) on day 5, when a peak of proliferation is known to occur in the DG after KA treatment (Carreira et al., 2010). The animals were sacrificed after at least 12 h, by transcardial perfusion.

### Neuroblast Migration

In order to investigate neuroblast migration after lesion, WT and hCAST mice were given either KA or SAL (6–9 animals per group) and sacrificed by transcardial perfusion, after 14 days (Figure 1G), when an increase in cell migration is known to occur in the DG after KA treatment (Carreira et al., 2015). Cell migration in the DG was then assessed by analyzing neuroblast staining with doublecortin (DCX), as described further in more detail.

### EdU Detection and Immunohistochemistry

Brains used for immunohistochemistry and EdU detection were obtained after transcardial perfusion of the mice with 0.9% NaCl and 4% paraformaldehyde. The brains were removed and kept in 4% paraformaldehyde overnight for further fixation and then dehydrated in 20% sucrose in phosphate buffer for at least one day, at 4°C. Coronal sections from the hippocampal region were cryosectioned (30 μm thick, in 6-series) using a CryoStar NX50 cryostat (Thermo Fisher Scientific, Waltham, MA, USA) and stored in an antifreeze solution (30% ethylene glycol and 30% glycerol in phosphate buffer), at 4°C. Free-floating brain sections from one of the series were labeled against BrdU, DCX, EdU or EdU/NeuN (neuronal nuclei), as previously described (Morte et al., 2013; Machado et al., 2015). EdU labeling was performed using a commercially available kit (Click-iT^®^ EdU Alexa Fluor^®^ 488 HCS Assay, Thermo Fisher Scientific, Waltham, MA, USA). After rinsing with 3% bovine serum albumin, the sections were permeabilized with 0.5% triton X-100 for 45 min, rinsed again and then incubated with a reaction cocktail (reaction buffer, CuSO4, Alexa Fluor 488 and reaction buffer additive) for 1 h, protected from light, at room temperature. For immunohistochemistry, sections were rinsed with PBS and then blocked for 1 h, at room temperature, in 5% blocking solution in 0.25% triton X-100, using normal horse serum. After blocking, the sections were kept with the primary antibodies—rat anti-BrdU 1:50 (AbD Serotec, Oxford, UK), goat anti-DCX 1:400 (Santa Cruz Biotechnology, Inc., Dallas, TX, USA), mouse anti-NeuN 1:200 (Merck Millipore, Billerica, MA, USA)—for 48 h, at 4°C. Sections were then rinsed in 2% blocking solution and incubated for 2 h, at room temperature and protected from light, with the correspondent Alexa Fluor-conjugated secondary antibodies—donkey anti-rat 488, donkey anti-goat 594 and donkey anti-mouse 594; 1:200 (Thermo Fisher Scientific, Waltham, MA, USA). For the labeling of BrdU, a DNA denaturation step with 1 M HCl for 20 min, at 65°C, was performed in the beginning. In the cases where NeuN was not labeled, nuclei were stained with 2 μg/ml Hoechst 33342 for 10 min, at room temperature. For the combination of EdU and NeuN, EdU labeling was performed, followed by the immunohistochemistry, starting with the blocking step. The entire procedure was done using an orbital shaker. After a final rinsing step, the sections were mounted in gelatin-coated slides, with DAKO fluorescence mounting medium.

### Analysis of Incorporation of BrdU and EdU

For the analysis of incorportation of the thymidine analogs, BrdU-positive and EdU-positive cells in the SGZ, the first layer of cells adjacent to the hilus, in the GZ or in the hilus of five central coronal sections of the hippocampal region were counted for each animal (Salazar-Colocho et al., 2008; Machado et al., 2015), directly under an epifluorescence microscope (Axio Imager Z2 microscope, Zeiss, Oberkochen, Germany). Cell counting was carried out in both upper and lower blades of the DG.

### DCX Immunoreactivity

DCX immunoreactivity in the DG was determined in images acquired in a laser scanning microscope (LSM710, Zeiss, Jena, Germany). The quantification of the DCX-positive area was performed in ImageJ (version 1.47v, National Institutes of Health, Bethesda, MD, USA), using a threshold analysis in five central coronal sections of the hippocampal region of each animal. This consisted in defining the optimal staining threshold and calculating the area labeled with DCX (Komitova et al., 2005; Machado et al., 2015).

### Neuronal Differentiation Analysis

For the quantification of newborn neurons, the percentage of cells labeled for both EdU and NeuN was determined in a total of up to 50 EdU-positive cells (Carreira et al., 2015) in the DG of eight animals per group, in images (orthogonal reconstructions of projections from 0.73 μm z-stacks) acquired in a LSM710 (Zeiss, Jena, Germany).

### FluoroJade C Staining

For FluoroJade C staining, coronal sections from the hippocampal region of WT and hCAST mice (3–4 animals per group) treated with KA for 24 h were mounted in gelatin-coated slides. After air-drying for at least 24 h, the sections were rinsed twice in distilled water, and then immersed for 5 min in 0.1% sodium hydroxide prepared in 80% ethanol, 1 min in 70% ethanol and rinsed twice for 2 min in distilled water. The slides with the sections were then transferred to a 0.06% potassium permanganate solution and left in an orbital shaker for 10 min. After another rinsing step, the slides were left with agitation and protected from light for 10 min in 0.0001% FluoroJade C prepared in 0.1% acetic acid. The sections were rinsed three more times for 1 min in distilled water and dried for 15 min at 60°C, after which they were immersed in xylene and coverslipped with DPX mounting medium. Images were acquired in an epifluorescence microscope (Axio Imager Z2 microscope, Zeiss, Oberkochen, Germany) and the percentage of staining in the GZ and the hilus measured using a threshold analysis in ImageJ (version 1.47v, National Institutes of Health, Bethesda, MD, USA; Carreira et al., 2010; Machado et al., 2015).

### Statistical Analysis

Data are expressed as means ± SEM. Statistical significance was determined using the Kruskal-Wallis test, with Dunn’s post-test, or the Mann-Whitney test, as indicated in the figure legends, using the GraphPad Prism 5 software (GraphPad Software, Inc., La Jolla, CA, USA). Differences were considered significant when *p* < 0.05.

## Results

### Short-Term Impairment in Novel Object Exploration after KA Treatment Is Prevented in Mice Overexpressing CAST

**Figure 2:**
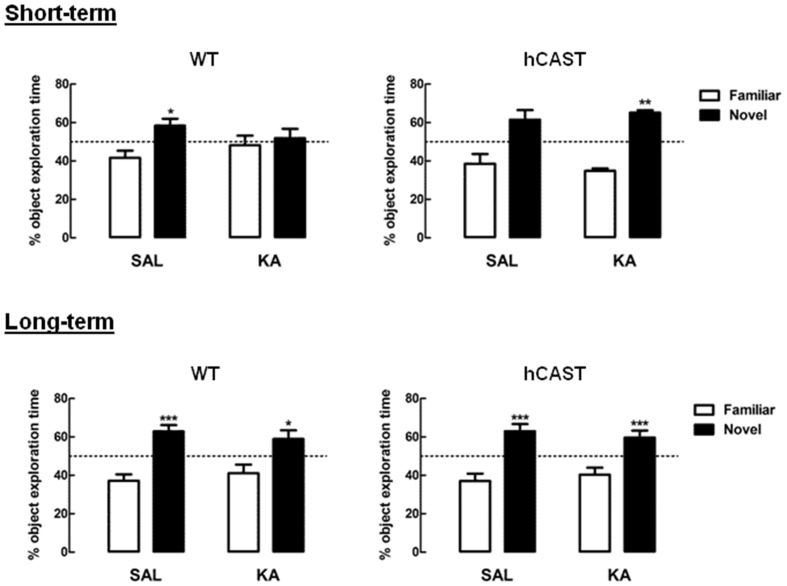
**KA-treated WT mice present short-term impairment in novel object exploration, but not KA-treated hCAST mice.** WT and hCAST mice behavior was tested on the 3rd (short-term) or 8th week (long-term) after treatment with either SAL or KA. Recognition memory was assessed by measuring the percentage of exploration time during the presentation of a novel object, in the OR test. Data are presented as means ± SEM of 3–5 animals per group (for short-term recovery) and 12–15 animals per group (for long-term recovery). Statistical significance was determined using the Kruskal-Wallis test (Dunn’s post-test), *p < 0.05, **p < 0.01 and ***p < 0.001, significantly different from familiar object.

To evaluate whether CAST overexpression would allow for a better and/or faster recovery after brain injury, we performed several cognitive behavioral tests on WT and hCAST mice, on the 3rd (short-term) or 8th (long-term) week after treatment with either KA or SAL. We first tested the animals for OR memory (3–5 animals per group for short-term recovery and 12–15 animals per group for long-term recovery). We observed that, for both time periods, WT mice treated with SAL spent significantly more time exploring the novel object (short-term recovery: 58.3 ± 3.7%, long-term recovery: 62.9 ± 3.3%) than the familiar object, that they had already explored previously (short-term recovery: 41.7 ± 3.7%, *p* < 0.05, long-term recovery: 37.1 ± 3.3%, *p* < 0.001). However, after KA treatment, only WT mice from the long-term recovery group were able to distinguish the novel object (58.9 ± 4.5%), from the familiar object (41.1 ± 4.5%, *p* < 0.05; Figure 2, left panels), indicating impairment in recognition memory in the short-term group. On the other hand, all mice overexpressing CAST spent more time exploring the novel object, in both short-term recovery (SAL, novel object: 61.5 ± 5.0%, familiar object: 38.5 ± 5.0%, *p* > 0.05; KA, novel object: 61.1 ± 1.3%, familiar object: 34.9 ± 1.3%, *p* < 0.01) and long-term recovery (SAL, novel object: 62.9 ± 3.8%, familiar object: 37.1 ± 3.8%, *p* < 0.001; KA, novel object: 59.7 ± 3.6%, familiar object: 40.3 ± 3.6%, *p* < 0.001; Figure 2, right panels).

### Long-Term Impairment in Spatial Memory for an Exact Location during the Water Maze Test after KA Treatment Is Prevented in Mice Overexpressing CAST

**Figure 3:**
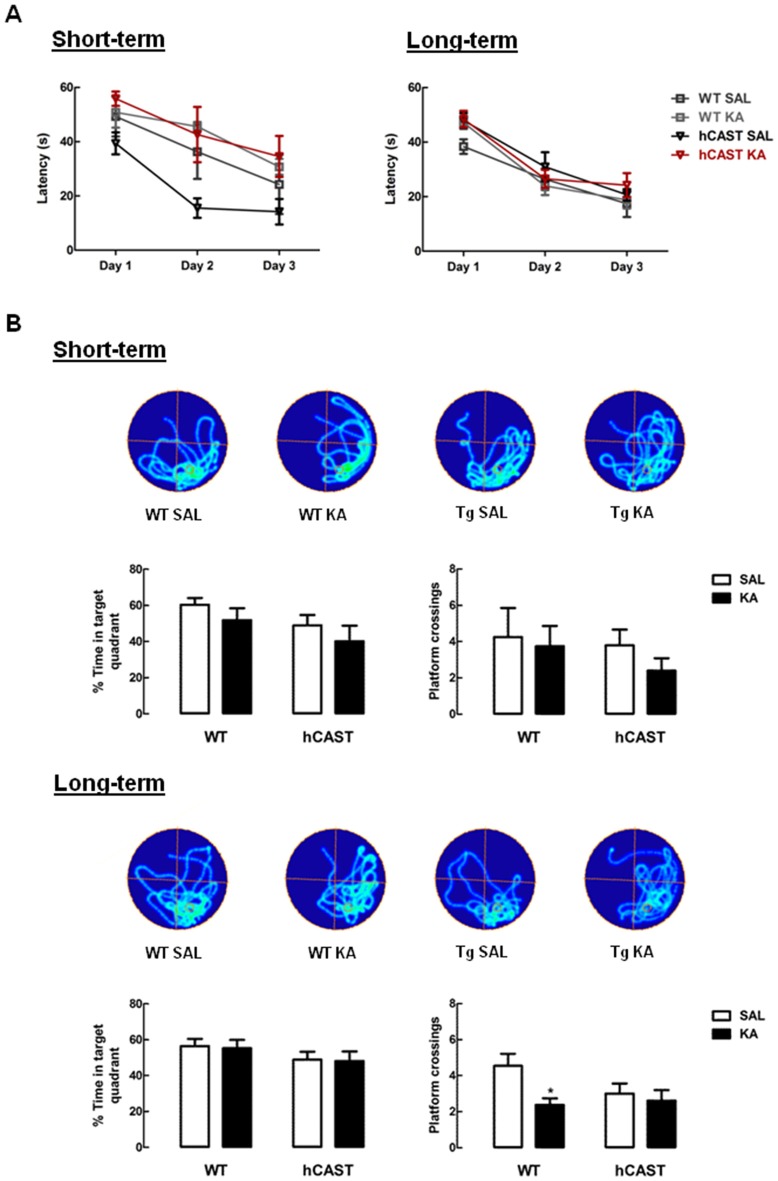
**Spatial memory for the exact platform location is impaired in KA-treated WT mice from the long-term recovery period.** WT and hCAST mice behavior was tested on the 3rd (short-term) or 8th (long-term) week after treatment with either SAL or KA. **(A)** Spatial learning was assessed by measuring the escape latency on the first 3 days of WM (four trials per day, latency of 60 s if the platform was not reached). **(B)** Spatial memory was assessed by measuring the percentage of time spent on the target quadrant and the number of crossings in the exact location the platform was during trials, on the last day of WM. Representative images of occupancy track plots for each group are shown in the top panels. Data are presented as means ± SEM of 4–5 animals per group (for short-term recovery) and 11–16 animals per group (for long-term recovery). Statistical significance was determined using the Kruskal-Wallis test (Dunn’s post-test), *p < 0.05, significantly different from SAL.

In order to study spatial learning and memory, the Morris WM test was performed (4–5 animals per group for short-term recovery and 11–16 animals per group for long-term recovery). During trials (Figure 3A), we observed that KA-treated mice in the short-term recovery period learned the platform location similarly to SAL on all days (Day 1, WT: 49.3 ± 7.3 s, hCAST: 39.4 ± 4.1 s; Day 2, WT: 36.3 ± 10.0 s, hCAST: 15.5 ± 3.6 s; Day 3, WT: 24.2 ± 10.9 s, hCAST: 14.2 ± 4.7 s, *p* > 0.05), as well as all KA-treated mice in the long-term recovery period (Day 1, WT: 38.4 ± 2.7 s, hCAST: 47.9 ± 2.7 s; Day 2, WT: 26.4 ± 3.9 s, hCAST: 30.9 ± 5.3 s; Day 3, WT: 17.4 ± 4.9 s, hCAST: 20.7 ± 2.1 s, *p* > 0.05). All mice showed improved spatial learning over the course of the trials.

After learning the platform location, the platform was removed and the spatial memory evaluated by calculating the percentage of time spent in the target quadrant (where the platform previously was) and by counting the number of crossings through the exact previous platform location. Time spent in the target quadrant was maintained with KA treatment in both time periods, by comparison with SAL (short-term recovery, WT: 60.4 ± 3.7%, hCAST: 49.0 ± 5.7%, *p* > 0.05; long-term recovery, WT: 56.4 ± 4.1%, hCAST: 49.0 ± 4.3%, *p* > 0.05; Figure 3B, left panels), indicating that all animals remembered the relative position of the platform. The number of platform crossings (Figure 3B, right panels), in turn, also seemed to be maintained with KA treatment in the short-term recovery period when comparing to SAL (WT: 4.3 ± 1.6, hCAST: 3.8 ± 0.9, *p* > 0.05). However, in the long-term recovery period, the number of platform crossings was significantly impaired in KA-treated WT mice (2.4 ± 0.4) by comparison with SAL treatment (4.5 ± 0.7, *p* < 0.05), indicating increased difficulty in remembering the exact platform location, which is not observed in mice overexpressing CAST (SAL: 3.0 ± 0.6, *p* > 0.05).

### Associative Fear Memory Is Preserved after KA Treatment

**Figure 4:**
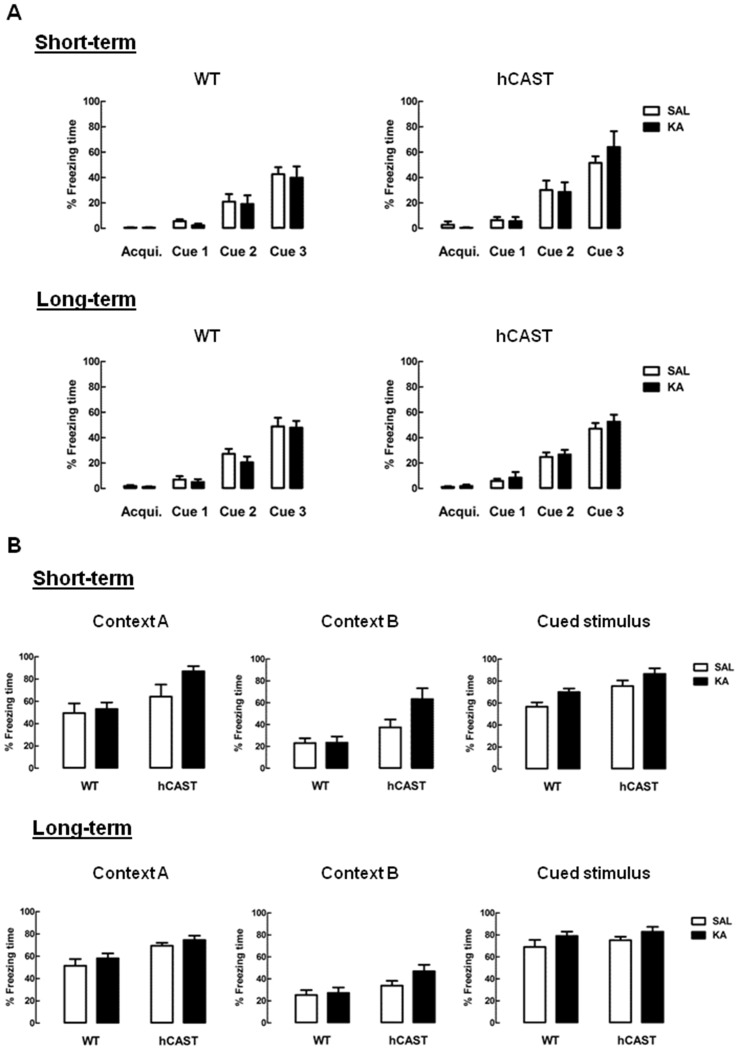
**Associative fear memory is preserved after KA treatment.** WT and hCAST mice behavior was tested on the 3rd (short-term) or 8th (long-term) week after treatment with either SAL or KA. **(A)** Associative learning was assessed by measuring the percentage of freezing time during FC trials, with the delivery of a footshock by the end of each cue tone. Acqui. (acquisition). **(B)** Associative memory to the context and cue tone were assessed by measuring the percentage of freezing time during the FC context test (context A) and cued test (cued stimulus), in a new context (context B). Data are presented as means ± SEM of 3–5 animals per group (for short-term recovery) and 9–16 animals per group (for long-term recovery). Statistical significance was determined using the Kruskal-Wallis test (Dunn’s post-test), *p* > 0.05 between treatments.

Lastly, associative learning and memory was evaluated, by performing the FC test (3–5 animals per group for short-term recovery and 9–16 animals per group for long-term recovery). By the end of the trials, on the first day, the animals successfully learned to associate the cue tone with a footshock. All animals spent nearly half the time of the last hearing of the tone freezing, both in the short-term recovery (WT SAL: 42.7 ± 5.4%, hCAST SAL: 51.5 ± 5.3%, *p* > 0.05) and in the long-term recovery (WT SAL: 48.9 ± 6.8%, hCAST SAL: 47.1 ± 4.5%, *p* > 0.05; Figure 4A).

When tested for associative memory to context (context A; Figure 4B, left panels), all KA-treated mice seemed to recognize the context similarly to SAL, both in the short-term recovery (WT: 48.9 ± 6.8%, hCAST: 64.2 ± 10.8%, *p* > 0.05) and in the long-term recovery (WT: 51.5 ± 6.0%, hCAST: 69.4 ± 2.6%, *p* > 0.05). When in the context meant for the cued test (context B; Figure 4B, middle panels), all hCAST mice from the short-term recovery group treated with KA spent over half the time freezing (SAL: 37.4 ± 7.2%, KA: 63.4 ± 9.8%, *p* > 0.05), while the percentage of freezing time was maintained low in WT mice (SAL: 23.1 ± 4.5%, KA: 23.5 ± 5.7%, *p* > 0.05). In the long-term recovery group, the percentage of freezing time was more similar to SAL in both WT and hCAST mice (WT: 25.4 ± 4.4%, hCAST: 33.8 ± 4.4%, *p* > 0.05). Moreover, all KA-treated mice seemed to maintain the associative memory to the cued stimulus by comparison with SAL, both in the short-term recovery (WT: 56.7 ± 3.9%, hCAST: 75.5 ± 5.2%, *p* > 0.05) and in the long-term recovery (WT: 69.1 ± 6.5%, hCAST: 75.1 ± 3.2%, *p* > 0.05; Figure 4B, right panels).

### CAST Overexpression Maintains Enhancement of Early Hippocampal Neurogenesis after KA Treatment

**Figure 5:**
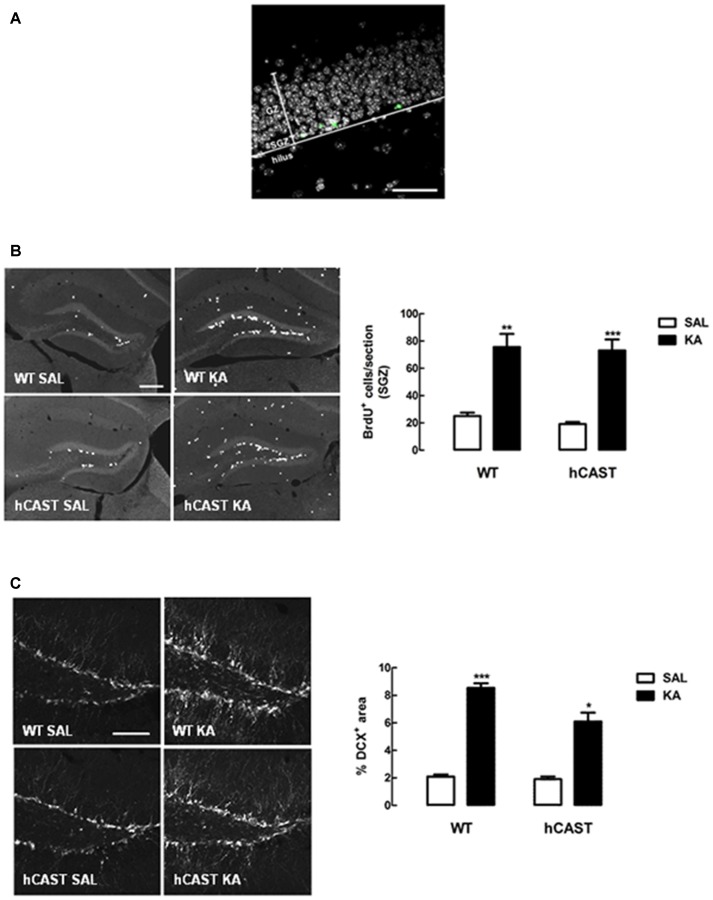
**KA treatment enhances NSC proliferation and neuroblast migration in the dentate gyrus (DG). (A)** Representative image of a section of the DG, showing the division into granular zone (GZ), subgranular zone (SGZ) and hilus. EdU in green and nuclei, stained for Hoechst 33342, in gray. Scale bar: 50 μm. **(B)** WT and hCAST mice were treated with either SAL or KA and were given BrdU on the day before sacrifice, to assess cell proliferation. Representative images from hippocampal brain sections for each group (left panels), showing BrdU-positive cells in white. Scale bar: 200 μm. BrdU-positive cells were counted in the SGZ of five central coronal sections of the hippocampal region for each animal (right panel). **(C)** WT and hCAST mice were treated with either SAL or KA and sacrificed after 14 days, to assess cell migration. Representative images from hippocampal brain sections for each group (left panels), showing migrating neuroblasts, labeled for doublecortin (DCX), in white. Scale bar: 100 μm. Percentage of DCX-positive area was determined in the DG of five central coronal sections of the hippocampal region for each animal (right panel). Data are presented as means ± SEM of 9–14 animals per group (for cell proliferation) and 6–9 animals per group (for cell migration). Statistical significance was determined using the Kruskal-Wallis test (Dunn’s post-test), *p < 0.05, **p < 0.01 and ***p < 0.001, significantly different from SAL.

In order to study the effects of CAST overexpression on the early stages of hippocampal neurogenesis after a brain insult, NSC proliferation and neuroblast migration were analyzed in the DG of adult mice (Figure 5A), after treatment with either SAL or KA (9–14 animals per group for cell proliferation and 6–9 animals per group for cell migration). Cell proliferation was assessed by incorporation of the thymidine analog BrdU into the DNA of dividing NSCs from the SGZ of the hippocampus. We observed that cell proliferation was greatly enhanced in mice overexpressing CAST after KA treatment (73.1 ± 8.0 cells/section), when compared to SAL-treated mice (19.2 ± 1.5 cells/section, *p* < 0.001), similarly to what was seen in WT mice (KA: 75.6 ± 9.7 cells/section, SAL: 25.0 ± 2.5 cells/section, *p* < 0.01; Figure 5B).

With regard to neuroblast migration, as assessed by DCX staining in the DG, we also observed a significant increase in KA-treated mice overexpressing CAST (6.1 ± 0.6%), when compared to SAL-treated mice (1.9 ± 0.2%, *p* < 0.05). Although cell migration in the DG of KA-treated WT mice (8.5 ± 0.3%) was also enhanced in comparison with SAL (2.1 ± 0.2%, *p* < 0.001), CAST overexpression seemed to attenuate this effect (Figure 5C).

To evaluate whether differences in neuronal death after lesion could be observed, WT and hCAST mice (3–4 animals per group) were sacrificed 24 h after KA treatment and FluoroJade C staining was performed. We observed that hCAST mice presented an amount of dying neurons similar to WT mice in the DG, both in the GZ (WT: 0.1 ± 0.1%, hCAST: 0.2 ± 0.0%, *p* > 0.05) and in the hilus (WT: 0.3 ± 0.2%, hCAST: 0.5 ± 0.1%, *p* > 0.05; Supplementary Figure 1).

### CAST Overexpression Affects Cell Differentiation in the DG Similarly to WT after KA Treatment

**Figure 6:**
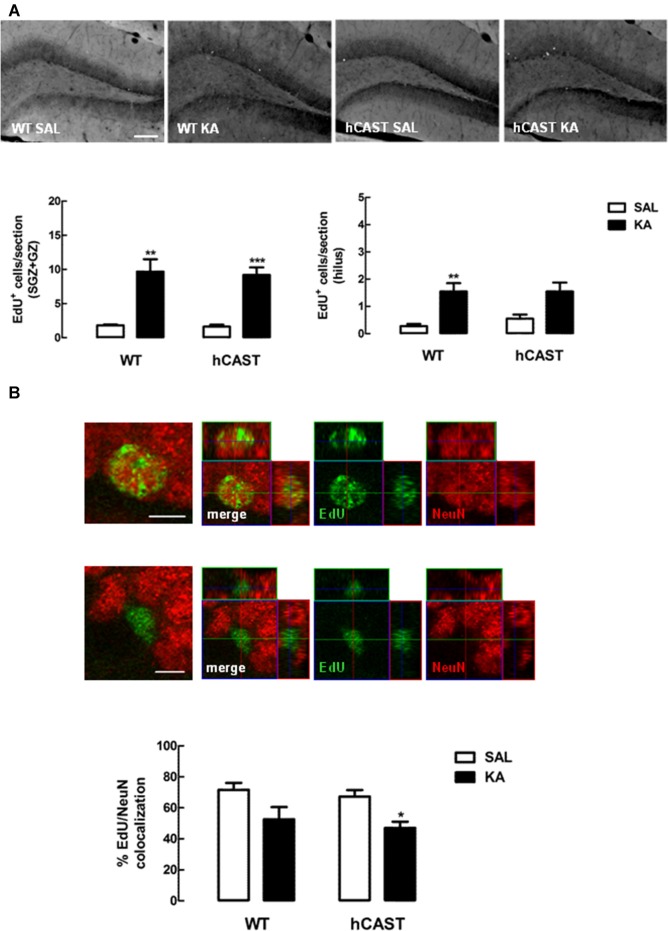
**CAST overexpression affects cell differentiation in the DG similarly to WT after KA treatment.** WT and hCAST mice were treated with either SAL or KA and sacrificed 8 weeks after EdU administration, to assess neuronal differentiation. **(A)** Representative images from hippocampal brain sections for each group (top panels), showing EdU-positive cells in white. Scale bar: 200 μm. EdU-positive cells were counted in the SGZ, GZ and hilus of five central coronal sections of the hippocampal region for each animal (bottom panels). **(B)** Representative images from hippocampal brain sections of EdU-positive/neuronal nuclei (NeuN)-positive and EdU-positive/NeuN-negative cells in the DG (top panels), showing cells labeled for EdU in green and cells labeled for NeuN in red. Scale bar: 5 μm. The percentage of cells labeled for both EdU and NeuN in the DG (bottom panel) was determined in a total of up to 50 EdU-positive cells. Data are presented as means ± SEM of eight animals per group. Statistical significance was determined using the Kruskal-Wallis test (Dunn’s post-test), *p < 0.05, **p < 0.01 and ***p < 0.001, significantly different from SAL.

Besides cell proliferation and migration, we were also interested in investigating differentiation of newborn cells after treatment with KA. For that purpose, mice were treated with the thymidine analog EdU (eight animals per group), and the number of EdU-positive cells remaining in the DG after 8 weeks was counted. We observed that the number of newborn cells still surviving in the DG was generally enhanced with KA-treatment in WT (SGZ + GZ: 9.7 ± 1.8 cells/section, hilus: 1.6 ± 0.3 cells/section) and hCAST (SGZ + GZ: 9.2 ± 1.1 cells/section, hilus: 1.6 ± 0.3 cells/section) mice, when compared to SAL treatment (SGZ + GZ, WT: 1.8 ± 0.1 cells/section, *p* < 0.01, hCAST: 1.6 ± 0.3 cells/section, *p* < 0.001; hilus, WT: 0.3 ± 0.1 cells/section, *p* < 0.01, hCAST: 0.6 ± 0.2 cells/section, *p* > 0.05; Figure 6A). When assessing neuronal differentiation specifically, by determining the percentage of those cells that colocalized with a neuronal marker (NeuN), we observed that treatment with KA seemed to reduce the amount of new neurons in the DG in WT mice (52.5 ± 8.0%) and in mice overexpressing CAST (47.0 ± 4.1%), when compared to SAL-treated animals (WT: 71.6 ± 4.4%, *p* > 0.05; hCAST: 67.2 ± 4.2%, *p* < 0.05; Figure 6B), suggesting decreased neuronal survival.

## Discussion

The realization that the adult mammalian brain is not an immutable organ and that new neurons are able to form and thrive in the neuronal circuitry has opened new doors for the putative treatment of neurodegenerative disorders and brain lesions. NSCs not only give origin to new neurons in the healthy brain, but are also known to increase the formation of new cells after lesion. NSCs can travel as neuroblasts into regions where neurons were lost as a consequence of brain damage, in what seems like an endogenous attempt to replace those lost neurons. However, this process is not very efficient, as most of the new cells die, fail to integrate the networks, or do it erroneously (Kaneko and Sawamoto, 2009; Ma et al., 2009). While cell transplantation into the sites of injury, for example, also seems promising, modulating endogenous neurogenesis would offer a less invasive approach for the treatment of brain damage. Calpains are proteases involved in neurodegeneration and their inhibition has been shown to be neuroprotective in several diseases (Saez et al., 2006), though their influence in neurogenesis is still not very clear. One of the approaches for the study of calpains is the modulation of their selective endogenous inhibitor, CAST. We had previously seen that the absence of CAST impairs NSC proliferation and neuroblast migration in the adult mouse brain (Machado et al., 2015), namely hippocampal neurogenesis, which has been implicated in learning and memory. Thus, we were interested in investigating whether overexpressing CAST would improve cognitive recovery after hippocampal injury, induced with KA, and its effects on post-injury neurogenesis. Finding a way to modulate calpain activity in order to help improve the outcome of brain injury would highly benefit patients with neurodegenerative diseases or other types of brain insults.

KA induces excitotoxicity and consequent cell death, by activating ionotropic receptors highly abundant in the hippocampus (Wang et al., 2005; Carta et al., 2014). The KA model of brain lesion is thus often used for research involving hippocampal damage, especially in studies of temporal lobe epilepsy (Levesque and Avoli, 2013). It is also a good model for investigating hippocampal injury and its consequences (Araújo et al., 2008; Carreira et al., 2010, 2015). Since the hippocampus is the main structure involved in learning and memory, it is not surprising that treatment with KA in rodents can induce cognitive impairment (Stubley-Weatherly et al., 1996), which has also been reported in patients with temporal lobe epilepsy (Hattiangady and Shetty, 2008). With this in mind, we were especially interested in verifying whether mice overexpressing CAST would show differences in cognitive recovery on the 3rd (short-term) or 8th week (long-term) after KA treatment. In the short-term group, neurons born a few days after the brain insult are still immature, while in the long-term group those neurons are already fully mature and integrated (Deng et al., 2010). We tested the mice for three different types of cognitive behavior: recognition memory (OR), spatial learning and memory (WM) and associative learning and memory (FC).

The OR test makes use of the natural exploratory behavior of rodents. In the presence of a familiar and a novel object, rats and mice will normally spend relatively more time exploring the novel one, in a process shown by several studies to implicate the hippocampus (Cohen and Stackman, 2015). It was thus not a surprise that KA treatment impaired recognition of the previously explored object in WT mice from the short-term recovery group. In the long-term group, however, this impairment seemed to be overcome, suggesting that the newly formed cells ultimately succeeded in restoring the networks necessary for OR. Interestingly, CAST overexpression prevented impairment after KA treatment even in the short-term group. Having in mind that in this group the newly formed neurons are still immature, it is unlikely that they were already able to restore lost connections. This might thus mean that the regions involved in recognition memory were less affected when CAST was overexpressed.

Spatial memory, in turn, has long been known to require the hippocampus, being one of the most affected by hippocampal lesions (Best et al., 2001; Martin and Clark, 2007). In a fundamental work by Richard Morris (Morris et al., 1982), for example, rats with hippocampi removed took longer to learn to reach an escape platform hidden in a pool, but not a visible platform, and also had more difficulty in distinguishing the exact previous escape location when the platform was removed from the pool. This indicates the importance of the hippocampal integrity in navigating through space using only distal environmental cues. In our study, we used this Morris WM test to investigate whether impairment in spatial memory was also seen in our model and if CAST overexpression could be beneficial. By the end of the trials, all animals were able to learn to reach the platform, and also spent a high percentage of time in the target quadrant when platform was removed. Moreover, in the short-term group, there seemed to be no differences in the number of crossings over the exact previous platform location. In the long-term group, however, the number of crossings was impaired in KA-treated WT mice in comparison with SAL, which was not seen in mice overexpressing CAST. Interestingly, certain neurons in the hippocampus, the place cells, have the peculiarity of firing only when the animal enters a specific position in space. Different place cells respond to different locations of the same environment, allowing the brain to create a spatial map of that particular environment (O’Keefe and Dostrovsky, 1971). Additionally, a study on the pattern of place-cell activity during a WM test in rats has shown that the percentage of place cells firing maximally was more than doubled near the platform when compared to other locations in the pool (Hollup et al., 2001). This indicates a great contribution of these cells to the correct mental positioning of the platform in this particular task. Our results may suggest a loss of place cells or correct place-cell activity in WT mice treated with KA, since they seemed to have only a general idea of the platform whereabouts, but failed to determine exactly where it was, as compared to SAL-treated mice. This notion is further corroborated by a study showing that seizure-induced hippocampal lesion can indeed affect the activity of place cells several weeks later, making them less precise and less stable (Liu et al., 2003). The existing place cells in mice overexpressing CAST seem therefore to be less affected by KA treatment, suggesting that calpain inhibition may also limit the seizure-induced damage of these cells.

The FC test was the last to be performed, since it was the most stressful for the animals. This test is based on the association of an aversive stimulus with a tone and with the context where it occurred, which is measured by the time the animal spends freezing, i.e., when the only observed movement is from breathing, a known indicative of fear in rodents (Blanchard and Blanchard, 1969; Wehner and Radcliffe, 2004). As with the other performed cognitive tests, the FC test also has the involvement of the hippocampus, but only in relation to context, being the response to the cued stimulus dependent on the integrity of the amygdala (Phillips and LeDoux, 1992). For that reason, during learning, we were expecting all mice to associate the cued tone with the aversive stimulus, with progressively more time spent freezing with further tone presentations. Indeed, by the last time the tone was played, all animals spent around half of the time freezing, in contrast with virtually no freezing that was observed when they were first placed in the chamber. On the following day, the animals were placed in the exact same context, without presentation of tone or footshock, and SAL-treated animals presented associative memory to this context, by presenting freezing behavior for most of the time spent inside the chamber, as expected. However, KA-treatment failed to impair the time spent freezing, which may be linked with the relative resistance of the used strain to KA-induced cell death (McKhann et al., 2003). Even though differences in associative memory might be too subtle for detection, freezing to the modified context seemed to be increased in KA-treated mice overexpressing CAST from the short-term recovery group, since they spent over half the time freezing. Unlike the other mice, that seemed to understand the context was different, these mice seemed to assimilate that they were in a similar situation as before. One interesting feature of the aforementioned place cells is that they can be involved in the mechanisms of pattern separation, the ability to discriminate between very similar memories, and pattern completion, the ability to recall a previous memory in full based only on some of the components that composed that memory (Sahay et al., 2011). If these mechanisms are not balanced, there is an alteration in the perception of the environment. For instance, if pattern completion is relatively enhanced, there is generalization of the current environment, which means that several contexts may be linked together, even if they present reduced similarities (Sahay et al., 2011). This imbalance may thus be what caused the tendency of KA-treated hCAST mice from the short-term group to present higher freezing in the modified context, since the small similarities between both contexts could have been enough for them to recall the aversive stimulus. A possible explanation for this may relate to the findings that young granule cells (immature neurons) seem to be involved with pattern separation and older granule cells with pattern completion (Nakashiba et al., 2012). Assuming that old neurons can be more resistant, and that this might be the case in KA-treated mice overexpressing CAST, relatively more younger ones would die (Spalding et al., 2013). Since KA enhances the early stages of neurogenesis, an increased number of younger granule cells is expected to exist in the hippocampus of mice from the short-term group. At first, the new cells do not appear to be enough to compensate for the relative higher number of older cells, though later on they seem to regain balance, since the differential behavioral response to the new context of hCAST mice treated with KA from the short-term recovery group is lost in the long-term group. On the other hand, assuming that in WT mice younger and older neurons are lost more or less equally in KA-treated WT mice, and since the balance between younger and older granule cells seems to be maintained, by observation of memory association to the new context, we suggest that the new young neurons may not be fully functional. Electrophysiology studies would help to evaluate if this is in fact the case. Lastly, like with learning, we were expecting, and observed, associative memory to the cue tone to be maintained in all animals, since the hippocampus does not seem to be involved in this process.

Furthermore, we wanted to evaluate whether CAST overexpression affected the modulation of post-injury hippocampal neurogenesis. Like with other types of brain damage, an enhancement of endogenous neurogenesis has also been reported after seizures. Independently of the model chosen to induce seizures in rodents, NSC proliferation is specially shown to be largely increased in the DG after just a few days (Parent et al., 1997; Gray and Sundstrom, 1998; Nakagawa et al., 2000; Hüttmann et al., 2003; Mohapel et al., 2004; Jessberger et al., 2007; Sierra et al., 2015). Interestingly, evidence of increased cell proliferation has also been observed in the hippocampus of patients with temporal lobe epilepsy (Blümcke et al., 2001; Crespel et al., 2005). In the model used by our group, the peak of cell proliferation in the DG was shown to occur on day 5 after seizure induction, with a significant increase in neuroblast migration after 14 days (Carreira et al., 2015), results that we were able to replicate in the present work. This heavy enhancement of early neurogenesis indicates that the microenvironment in the hippocampus not long after seizures is extremely pro-neurogenic, which makes it difficult for changes due to altered levels of calpain activity to be observed. Nonetheless, since increased calpain activity can impair NSC proliferation and migration of neuroblasts, it is important to exclude whether, in this case, calpain inhibition, by overexpression of CAST, increases cell proliferation and migration to alarming levels. An even more extreme rise in cell proliferation could originate unwanted cellular masses of undifferentiated cells, and more migrating cells could also mean more cells migrating into ectopic regions, interfering with the normal neuronal networks (Parent et al., 2006).

Regarding cell proliferation, the enhancement observed after KA treatment was similar in WT mice and in mice overexpressing CAST. Curiously, however, while the increase in neuroblast migration in KA-treated WT mice was around 4-fold, it dropped to 3-fold with the overexpression of CAST. Brain damage is closely associated with an increase in calpain activity, and it is known that calpain inhibition can be neuroprotective (Saez et al., 2006). This means that the fact that CAST was already overexpressed when seizures were induced may have attenuated the damage. Even though the amount of dying neurons in the DG 24 h after KA treatment seems similar to WT in a preliminary study by our group, the surrounding circuitry may still be more preserved with CAST overexpression, or different types of neurons may be affected. Less damage could therefore also mean a less dramatic increase in neurogenesis. In the long term this could be favorable, proven that more cells would integrate correctly in the existing networks. Cell proliferation in the DG was shown by another group to substantially rise even after less severe seizures, with no further increase with seizure severity (Mohapel et al., 2004). This might explain why we observed a similar increase in cell proliferation even with the overexpression of CAST.

This increase in neurogenesis after lesion, however, is only efficient if the new cells ultimately differentiate, survive and integrate the neuronal circuitry correctly. After seizures, this is usually not the case. Despite the enhancement in the early stages of neurogenesis, most of the new cells end up differentiating into astrocytes, with relatively less neurons surviving 1–2 months after seizures (Hattiangady and Shetty, 2010; Carreira et al., 2015; Sierra et al., 2015). Moreover, several newly formed neurons resembling granule cells are found ectopically in the hilus, and have also been reported to occur in patients with temporal lobe epilepsy (Parent et al., 1997, 2006). These ectopic granule-like hilar cells are hyperexcitable, contributing to a disruption of the existing neuronal networks and possibly aggravating the outcome of the disease (Dashtipour et al., 2001; Cameron et al., 2011). Our findings with WT mice are consistent with what has been reported. After a 2-month period, there were significantly more new cells found in the hilus, SGZ and GZ of the DG of mice treated with KA. Though not significant, the percentage of new neurons seemed to be decreased in these mice, which is in accordance with the aforementioned reduced survival of new neurons after seizures. The overall increase in the number of new cells may thus be justifiable by the increased differentiation into astrocytes that occurs after seizures. The results obtained with hCAST mice were very similar, indicating that suppression of calpain activity provided by CAST overexpression is not enough to compensate for the impairment of neuronal differentiation in the DG. Although calpain inhibition was shown to increase neuronal differentiation *in vitro*, in a cell line from fetal NSCs (Santos et al., 2012), we cannot exclude that *in vivo* and in the developed brain other mechanisms may compensate for the decrease in calpain activity under a certain threshold, or even help in maintaining levels of calpain activity too elevated, especially in a pathologic environment. In this case, additional factors, such as nitric oxide from inflammatory origin, may also contribute for neuronal impairment (Carreira et al., 2015). This multifactorial feature of brain pathology must thus be taken into account when developing new strategies for brain repair. Nonetheless, even though the percentage of neurons formed after seizures is not ameliorated by CAST overexpression, the ones that thrive may still be more functional and better integrated in the hippocampal neuronal networks.

Overall, the fact that CAST was already overexpressed prior to lesion may have masked potential benefits of calpain inhibition on post-injury neurogenesis. Moreover, the fact that the absence of CAST and, consequently, increased calpain activity, can impair hippocampal neurogenesis, does not necessarily mean that reduced or lack of calpain activity can enhance it. In conclusion, even though reduced calpain activity does not seem to be able to enhance hippocampal neurogenesis, it could potentially be able to prevent the impairment in the formation of new neurons after injury, given that the levels of calpain activity could be reduced under a certain threshold. Strategies to reduce the harmful effects of the pathologic environment, such as controlling neuroinflammation, could also help boost the outcome of calpain inhibition in the survival of new neurons. Moreover, these observations are in regard to hippocampal neurogenesis. Cells derived from the subventricular zone may hold more promise for the enhancement of endogenous neurogenesis with calpain inhibition, encouraging further research involving these cells and calpain activity in the field of brain damage.

## Author Contributions

VMM: conception and design of the work, acquisition, analysis, interpretation of data, drafting of manuscript; ASL: design and acquisition; CF and RF: acquisition; CMC: conception and design of the work; IMA: conception and design of the work, interpretation of data, drafting of manuscript. All authors revised and approved the final version of the manuscript.

## Funding

This work was supported by the Foundation for Science and Technology (FCT, Portugal), COMPETE and FEDER (grants PTDC/SAU-NMC/112183/2009, UID/NEU/04539/2013 and UID/BIM/04773/2013). VMM and ASL were supported by FCT (fellowships SFRH/BD/78050/2011 and SFRH/BD/79308/2011).

## Conflict of Interest Statement

The authors declare that the research was conducted in the absence of any commercial or financial relationships that could be construed as a potential conflict of interest. The reviewer DT and handling Editor declared their shared affiliation, and the handling Editor states that the process nevertheless met the standards of a fair and objective review.

## Abbreviations

BrdU: 5-bromo-2′-deoxyuridine
CAST: calpastatin
DCX: doublecortin
DG: dentate gyrus
EdU: 5-ethynyl-2′-deoxyuridine
FC: fear conditioning
GZ: granular zone
hCAST: mice overexpressing calpastatin
KA: kainic acid
NeuN: neuronal nuclei
NSC: neural stem cell
OR: object recognition
SAL: saline
SGZ: subgranular zone
WM: water maze
WT: wild type.
