# Supplementary material for: Calpastatin Overexpression Preserves Cognitive Function Following Seizures, While Maintaining Post-Injury Neurogenesis
Source: Front Mol Neurosci. 2017 Mar 23;10:60. doi: 10.3389/fnmol.2017.00060 (PMC5362605; doi:10.3389/fnmol.2017.00060)
Supplement: Supplementary file 1 [file Image_1.pdf]

Supplementary Material

Calpastatin overexpression preserves cognitive function following seizures, while maintaining post-injury neurogenesis

Vanessa M. Machado, Ana Sofia Lourenço, Cláudia Florindo, Raquel Fernandes, Caetana M. Carvalho, Inês M. Araújo*

*** Correspondence:**
Inês M. Araújo, PhD
imaraujo@ualg.pt
Department of Biomedical Sciences and Medicine, University of Algarve
Faro, Portugal

1 Supplementary Figures

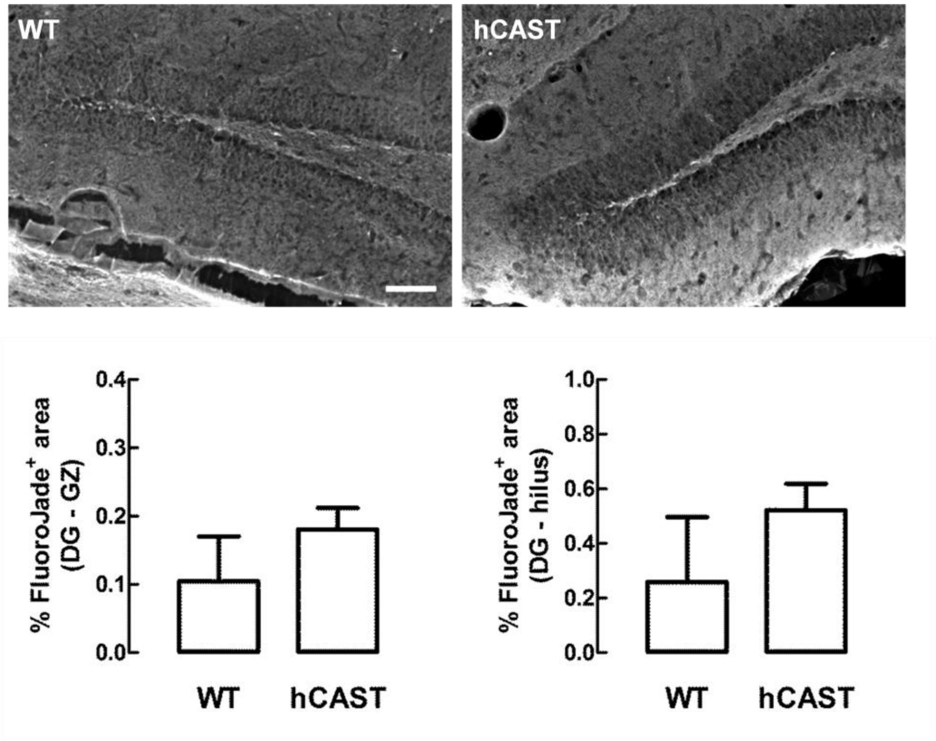

Supplementary Figure 1 - Neuronal death in the DG after KA treatment is maintained with CAST overexpression. WT and hCAST mice were treated with KA and sacrificed after 24 h. FluoroJade C staining was performed, in order to assess neuronal death. Representative images are shown in the top panels, with degenerating neurons in white, in the DG. Scale bar: 50 µm. Percentage of FluoroJade-positive area in the hilus and the granular zone are shown in the bottom panels. Data are presented as means ± SEM of 3-4 animals per group. Statistical significance was determined using the Mann-Whitney test, $p>0.05$.
